# Evaluation of Antioxidant and Antimicrobial Activities and Phenolic Profile for *Hyssopus officinalis, Ocimum basilicum* and *Teucrium chamaedrys*

**DOI:** 10.3390/molecules19055490

**Published:** 2014-04-28

**Authors:** Laurian Vlase, Daniela Benedec, Daniela Hanganu, Grigore Damian, Ioan Csillag, Bogdan Sevastre, Augustin C. Mot, Radu Silaghi-Dumitrescu, Ioan Tilea

**Affiliations:** 1 Department of Pharmaceutical Technology and Biopharmaceutics, Iuliu Hatieganu University of Medicine and Pharmacy, 12 I. Creanga Street, Cluj-Napoca 400010, Romania; E-Mail: laurian.vlase@umfcluj.ro; 2 Department of Pharmacognosy, Iuliu Hatieganu University of Medicine and Pharmacy, 12 I. Creanga Street, Cluj-Napoca 400010, Romania; 3 Department of Physics, Babes-Bolyai University, 1 M. Kogalniceanu Street, Cluj-Napoca 400084, Romania; E-Mails: grigore.damian@phys.ubbcluj.ro (G.D.); bapo25@yahoo.it (I.C.); 4 Department of Physiopathology, University of Agricultural Sciences and Veterinary Medicine, 3-5 Mănăştur Street, Cluj-Napoca 400372, Romania; E-Mail: bogdan.sevastre@usamvcluj.ro; 5 Department of Chemistry and Chemical Engineering Babes-Bolyai University, 11 A. Janos Street, Cluj-Napoca 400028, Romania; E-Mails: augustinmot@chem.ubbcluj.ro (A.C.M.); rsilaghi@chem.ubbcluj.ro (R.S.-D.); 6 Family Medicine, Department M3 Clinical Sciences Internal Medicine, University of Medicine and Pharmacy, 38 G. Marinescu Street, Târgu Mures 540139, Romania; E-Mail: ioan.tilea@umftgm.ro

**Keywords:** antioxidant and antimicrobial activities, *Hyssopus officinalis*, *Ocimum basilicum*, *Teucrium chamaedrys*, polyphenols

## Abstract

This study was designed to examine the *in vitro* antioxidant and antimicrobial activities and to characterize the polyphenolic composition of the ethanolic extracts of *Hyssopus officinalis, Ocimum basilicum* and *Teucrium chamaedrys*. Qualitative and quantitative analysis of the major phenolic compounds were conducted using high-performance liquid chromatography coupled to mass spectrometry (HPLC-MS). The total polyphenols, caffeic acid derivatives and flavonoids content was spectrophotometrically determined. The phenolic profile showed the presence of phenolic acid derivatives (caftaric, gentisic, caffeic, *p*-coumaric, chlorogenic and ferulic acids), flavonoid glycosides (rutin, isoquercitrin and quercitrin) and free flavonoid aglycons (luteolin, quercetin), in different concentrations. DPPH radical scavenging assay, Trolox equivalent antioxidant capacity (TEAC) method, hemoglobin ascorbate peroxidase activity inhibition (HAPX) assay, and electron paramagnetic resonance (EPR) radicals detection were employed, revealing several aspects of the antioxidant activities of these species. The antimicrobial tests were performed using the disk diffusion assay. These extracts contained a large amount of the polyphenolic compounds (77.72, 175.57, and 243.65 mg/g, respectively), and they showed a good antioxidant activity, as witnessed by a number of methods. *T. chamaedrys* had a high antimicrobial activity. Besides their antioxidant activity, the antimicrobial effect of these extracts confirms the biological activities of these herbal medicinal products.

## 1. Introduction

The flora of Romania comprises around 33 genera and more than 130 species and several varieties and subspecies of the *Lamiaceae* family [1]. This family has an almost cosmopolitan distribution from temperate to tropical regions but is found primarily in the Mediterranean basin [2]. Generally, the aromatic plants and spices of the *Lamiaceae* are rich in polyphenolic compounds and a large number of them are well known for their antioxidant properties [1,2,3,4]. In this regard, *Hyssopus officinalis*, *Ocimum basilicum* and *Teucrium chamaedrys* are very important members of this family for their medicinal value [3,4]. Natural antioxidants are being extensively studied for their ability to protect organisms and cells from damage caused by oxidative stress. Herbs and spices are, in general, harmless sources for obtaining natural antioxidants. There is an increasing demand to evaluate the antioxidant properties of the herbal extracts and in the last years, the attention has been focused on the antioxidant products from natural sources [2,3,4].

*T. chamaedrys* (wall germander) is a species of the genus *Teucrium.* This genus includes five species and a few subspecies and varieties in the spontaneous flora of Romania [1]. The aerial parts have been used as bitter, astringent, digestive, antispasmodic and anti-inflammatory agents, for centuries. Due to its pharmacological effects, this plant is widely used in traditional medicine, in the treatment of digestive disorders, coughs, asthma, abscesses, conjunctivitis and cellulite [4,5,6]. Several studies about antimicrobial, spasmolytic, antiviral, antioxidant and anti-inflammatory effects of the different species of *Teucrium* have also been reported in the literature [7,8,9,10,11,12].

*H. officinalis* and *O. basilicum* are some of the most important pharmaceutical herbs extensively cultivated in Romania [1]. Hyssop is a typical xerophyte and is well adapted to drought and low input conditions [1,13]. Despite having a bitter taste, hyssop is used as a food flavor and in sauce formulations. This herb has been used traditionally for medicinal purposes, for antispasmodic, stomachic, antifungal and cough treatments; generally, these therapeutic uses and health benefits of hyssop are largely based on folklore rather than on scientific substantiation [13,14,15]. A literature review on the chemical and biological aspects of the plant indicates that the main constituents of hyssop include several polyphenolic compounds and essential oil [13,16,17]. The extracts and the essential oil isolated from hyssop showed moderate antioxidant and antimicrobial activity together with antifungal and insecticidal antiviral properties, *in vitro* [13,18,19,20]. Animal model studies indicated myorelaxant, antiplatelet, and α-glucosidase inhibitory activities for this plant [13]. The essential oil is mainly used for flavouring and food preservation and for phytotherapeutic uses [21].

*Ocimum*, named basil, is another member of the *Lamiaceae* family, known as an aromatic and medicinal plant that has been used traditionally in the treatment of headaches, coughs, constipation, warts, worms and kidney malfunctions [1,22]. It has a long history as culinary herb adding a distinctive flavor to many foods, which can be attributed to its foliage. *O. basilicum* extracts have been shown to contain poly phenolic compounds, vitamins and essential oils that possess insecticidal, nematicidal, fungistatic, antimicrobial, and anti-inflammatory properties. In view of its therapeutic potential and its importance as a culinary base ingredient, basil deserves further scientific attention [23,24,25,26,27]. *H.* *officinalis* and *O.* *basilicum* are used in everyday life in various medicinal, cosmetics and food items [13,26,28]. *T. chamaedrys* is only used in traditional medicine [4,7]. These species produce valuable secondary metabolites, with notable therapeutical properties. It is mandatory to increase understanding of the biological activities of these species. In addition, further comprehensive studies of polyphenolic compounds are essential [13,27]. It was employed a rapid, highly accurate and sensitive HPLC method assisted by MS detection for the simultaneous determination of polyphenols in the plant extracts [28,29,30,31,32]. Considering these aspects, the natural products continue to be an important source of medicines and supplementary health products which represent a challenge to science due to their various properties, including chemical diversity, and variable compositions.

The aim of this work was to analyze the chemical composition of the ethanolic extracts of *H. officinalis*, *O. basilicum* and *T. chamaedrys* from Romania and to investigate their antioxidant and antimicrobial properties, for a better characterization and exploitation of these natural products.

## 2. Results and Discussion

### 2.1. HPLC Analysis of Polyphenols

**Figure 1:**
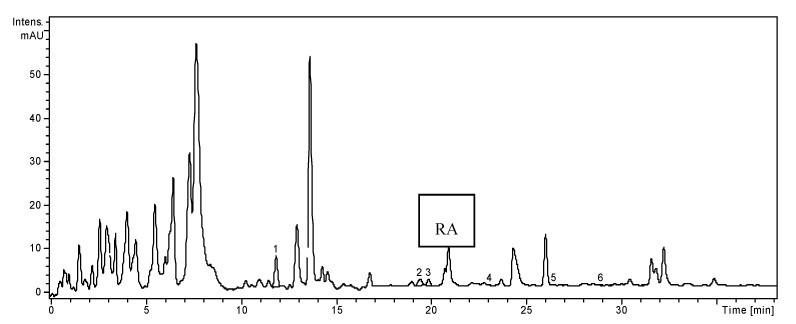
HPLC chromatogram of *H. officinalis* sample.

Notes: Chromatographic conditions were as given in the Experimental section. The identified compounds: 1, Ferulic acid; 2, Isoquercitrin; 3, Rutin; 4, Quercitrin; 5, Quercetin; 6, Luteolin ; RA, rosmarinic acid.

**Figure 2:**
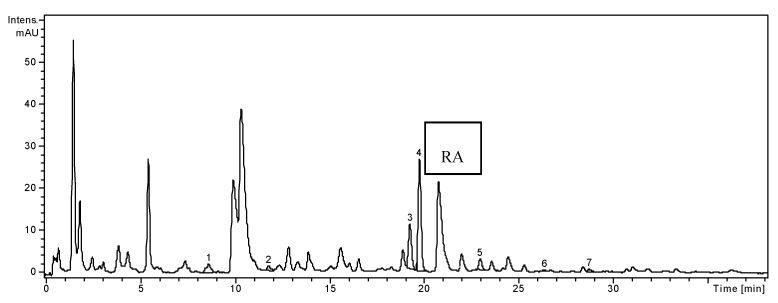
HPLC chromatogram of *O.basilicum* sample.

Notes: Chromatographic conditions were as given in the Experimental section. The identified compounds: 1, *p*-Coumaric acid; 2, Ferulic acid; 3, Isoquercitrin; 4, Rutin; 5, Quercitrin; 6, Quercetin; 7, Luteolin.; RA, rosmarinic acid.

**Figure 3:**
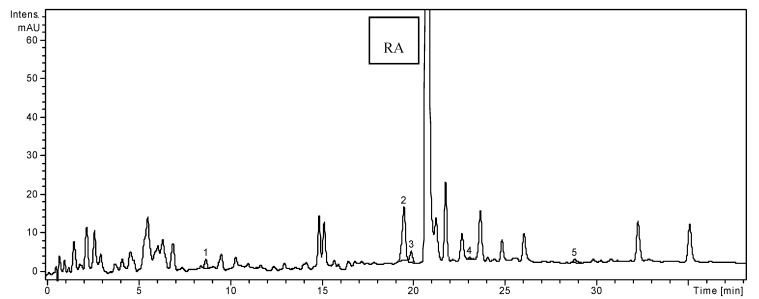
HPLC chromatogram of *T. chamaedrys.*

Notes: Chromatographic conditions were as given in the Experimental section. The identified compounds: 1, *p*-Coumaric acid; 2, Isoquercitrin; 3, Rutin; 4, Quercitrin; 5, Luteolin; RA, rosmarinic acid.

HPLC coupled with MS is a very powerful analytical technique, due to its high sensitivity and the structural information that can be obtained about the analytes. A high-performance liquid chromatographic (HPLC) method has been developed for the determination of 19 phenolic compounds: eight phenolic acids, four quercetin glycosides, and seven flavonol and flavone aglycones, from plant material. The applicability of the proposed analytical method and the qualitative and quantitative determination of the standard phenolic compounds have already been verified [28,29,30,31]. The method allows a simultaneous analysis of different classes of polyphenols by a single pass column (the separation of all examined compounds was carried out in 35 min). The quantitative determination was performed using the external standard method. The concentrations of identified polyphenolic compounds in all analyzed samples are presented in Table 1. They were shown in the order of their retention time. The HPLC chromatograms of *H. officinalis*, *O. basilicum* and *T. chamaedrys* samples are presented in Figure 1, Figure 2 and Figure 3.

**Table 1:** The polyphenolic compounds content in the studied species (µg/g plant material).

Polyphenolic Compounds	*m/z* Value	t_R_ ± SD (min)	*H. officinalis*	*O. basilicum*	*T. chamaedrys*
Caftaric acid	311	3.54 ± 0.05	<0.2	<0.2	NF
Gentisic acic	179	3.52 ± 0.04	<0.2	<0.2	<0.2
Caffeic acid	179	5.60 ± 0.04	<0.2	<0.2	NF
Chlorogenic acid	353	5.62 ± 0.05	<0.2	<0.2	<0.2
*p*-Coumaric acid	163	9.48 ± 0.08	<0.2	21.26 ± 0.63	25.68 ± 0.33
Ferulic acid	193	12.8 ± 0.10	36.92 ± 1.00	7.09 ± 0.07	NF
Isoquercitrin	463	19.60 ± 0.10	32.78 ± 0.23	179.19 ± 1.93	524.8 ± 2.75
Rutin	609	20.20 ± 0.15	21.93 ± 0.72	425.71 ± 2.15	85.42 ± 0.9
Rosmarinic acid	360	20.8 ± 0.10	<0.2	<0.2	<0.2
Quercitrin	447	23.64 ± 0.13	4.02 ± 0.07	50.39 ± 0.85	18.52 ± 0.49
Quercetin	301	26.80 ± 0.15	1.79 ± 0.03	3.39 ± 0.09	NF
Luteolin	285	29.10 ± 0.19	2.25 ± 0.03	6.06 ± 0.05	20.42 ± 0.47

Note: NF - not found, below limit of detection. Values are the mean ± SD (n = 3).

Caftaric, gentisic, caffeic, chlorogenic and *p*-coumaric acids were identified in the ethanolic extract of *H. officinalis*, but only ferulic acid was quantified (36.92 ± 1.0 µg/g). The most abundant phenolic acids reported by Greek authors for indigenous *H. officinalis* were considered to be ferulic acid (13.2 mg/100 g) and caffeic acid (6.5 mg/100 g) [13]. Three flavonoid glycosides, isoquercitrin, rutin and quercitrin, and two flavonoid aglycone, quercetin and luteolin were found in hyssop. Isoquercitrin was the flavonoid found in the largest amount (32.78 ± 0.23 µg/g) (Table 1). Chinese and Greek authors reported the presence of other derivates of catechin, apigenin, diosmin and acacetin in hyssop [13].

In the ethanolic extract of *O. basilicum*, two hydroxycinnamic acid derivates, namely ferulic acid and *p*-coumaric acid were identified and quantified (Table 1). Caftaric, gentisic, caffeic acid and chlorogenic acids were also identified in the basil extract, but they were in too low concentration to be quantified. Three flavonoid glycosides, isoquercitrin (quercetin 3-glucoside), rutin (quercetin-3-*O*-rutinoside) and quercitrin (quercetin 3-rhamnoside) were identified and quantified (Table 1), considering the flavonoid standards used. Rutin was the compound found in the largest amount (425.71 ± 2.15 µg/g), followed by isoquercitrin (179.19 ± 1.93 µg/g) and quercitrin (50.39 ± 0.85 µg/g). Two free flavonoid aglycons, *i.e.*, quercetin and luteolin, were found in small quantities (3.39 ± 0.09, and 6.06 ± 0.05 µg/g, respectively). Gentisic, caffeic, ferulic (4%) and p-hydroxybenzoic acids were determined in the extract of Greek basil [8].

In the ethanolic extract of *T. chamaedrys* two hydroxycynnamic acid derivates (chlorogenic and *p*-coumaric acids), one dihydroxybenzoic acid (gentisic acid), one flavone (luteolin) and three flavonoid glycosides (isoquercitrin, rutin and quercitrin) were detected (Table 1). The extract of *T. chamaedrys* was the richest in isoquercitrin (524.8 ± 2.75 µg/g), *p*-coumaric acid (25.68 ± 0.33 µg/g) and luteolin (20.42 ± 0.47 µg/g), compared to the other two samples. Gallic, caffeic, ferulic acids, apigenin, and quercetin hydrated were quantified in the extract of *T. chamaedrys* from Greece [8].

Considering the 19 standard compounds used in this study, some other peaks were not identified. Nevertheless, in all three chromatograms (Figure 1, Figure 2 and Figure 3), a signal can be seen at 20.8 min. The MS spectrum of this compound corresponds to rosmarinic acid and it was previously reported by our research group in other vegetal extracts [32]. Thus, the rosmarinic acid was included in Table 1, as qualitative data (no quantification for it).We analyzed the polyphenols from three *Lamiaceae* species: *O. basilicum*, *H. officinalis* and *T. chamaedrys.* The simultaneous determination of wide range of polyphenolic compounds was performed using a rapid, highly accurate and sensitive HPLC method assisted by mass spectrometry detection [28,29,30,31,32]. The comparative study showed significant differences in the composition of the three investigated species, especially quantitative. A one-way ANOVA test applied on the concentrations values of the identified compounds listed in Table 1 showed that there is a highly significant difference between these three extracts (*p* < 0.001).

### 2.2. Determination of Phenolic Compounds Content

Free radical damage contributes to the etiology of many chronic health problems such as cardiovascular and inflammatory disease, cancer, *etc.* Polyphenolic compounds function as reducing agents, free radical scavengers, and quenchers of singlet oxygen. In addition, flavonoids and phenolic acids components play important roles in the control of cancer and other human diseases, reducing the risk of cardiovascular disease, as antioxidants. Due to their importance in plants and human health, it would be useful to know the concentration of the phenolic compounds and biological activities that could indicate their potentials as therapeutic agents [2,31,33].

The results of the amount of total phenolic contents (TPC) and content of flavonoids and caffeic acid derivatives in *H. officinalis*, *O. basilicum* and *T. chamaedrys* extracts are given in Table 2. Thus, TPC values were expressed as gallic acid equivalent (mg GAE/g plant material). The phenolic acids contents were expressed as caffeic acid equivalent (mg CAE/g plant material). The calculation of total flavonoid content of plant extracts was carried out using the standard curve of rutin and presented as rutin equivalents (mg RE/g plant material).

**Table 2:** The content of total polyphenols, flavonoids and caffeic acid derivatives in the extracts.

Samples	TPC (mg GAE/g Plant Material)	Flavonoids (mg RE/g Plant Material)	Caffeic Acid Derivatives (mg CAE/g Plant Material)
*H. officinalis*	77.72 ± 1.83	1.30 ± 0.10	9.25 ± 0.75
*O. basilicum*	175.57 ± 2.43	6.72 ± 0.19	12.11 ± 0.39
*T. chamaedrys*	243.65 ± 3.46	9.75 ± 0.25	12.51 ± 0.20

Each value is the mean ± SD of three independent measurements. GAE: Gallic acid equivalents; RE: rutin equivalents; CAE: caffeic acid equivalents.

The extract of *T. chamaedrys* contained the highest amount of polyphenolic, flavonoidic compounds,and caffeic acid derivates (243.65 ± 3.46, 9.75 ± 0.25, and 12.51 ± 0.20 mg/g respectively), followed by the extract of *O. basilicum* (175.57 ± 2.43, 6.72 ± 0.19, and 12.11 ± 0.39 mg/g respectively). The lowest total polyphenols, flavonoids and caffeic acid derivatives concentration was measured in the extract of *H. officinalis* (77.72 ± 1.83, 1.30 ± 0.10, and 9.25 ± 0.75 mg/g respectively). A one-way ANOVA test was employed for the values found in Table 2 and the statistical results (*p* < 0.001) sustained the highly significant difference between the three extracts in terms of their total polyphenolic, caffeic acids derivatives and total flavonoid content. Concerning the content of polyphenols, the extract of *Teucrium* species from Romania was richer than the extracts of *T. chamaedrys* from Serbia, Montenegro and Turkey (168.46, 159.84, and 69.75 mg/g, respectively) [5,6,7,10]. The same species had a lower concentration of flavonoids than *T. chamaedrys* from Serbia and Montenegro (16.67 ± 0.21 mg/g; 61.80 ± 0.18 mg/g) [5,6]. The results obtained for other samples of *O. basilicum* from Romania using various extractions emphasized a range of values for total polyphenolic compounds (from 9.12% to 20.33%), flavonoids (from 0.35% to 1.24%) and caffeic acid derivates (from 0.61% to 2.02%). The results obtained for our sample exceeded these limits (except flavonoids) [28]. Comparing the polyphenolic content, the methanolic extract obtained from Iranian *H. officinalis* var. *angustifolius* was richer than the ethanolic extract obtained from Romanian hyssop (90 mg/g and 77.72 mg/g, respectively) [13]. In other Romanian samples, the amount of caffeic acid derivatives was highest than our sample (1.69% and 0.9%, respectively) [13,16,17]. The presence of active principles depends on a number of factors including the plant species, genetic factors, geographical location, differences in growth, the type of soil, the time and season of harvest, the way the herb is prepared, drying, and storage [28]. The result of the present study suggests that these plant medicinals especially *T. chamaedrys* and *O. basilicum* may be considered a potential source of polyphenols.

### 2.3. Antioxidant Activity

The antioxidant activity of the ethanolic extracts of *H. officinalis*, *O. basilicum* and *T. chamaedrys* was evaluated using the DPPH bleaching method, Trolox equivalent antioxidant capacity (TEAC) assay, hemoglobin ascorbate peroxidase activity inhibition (HAPX) assay, and an electron Paramagnetic Resonance (EPR) spectroscopy method (Table 3).

**Table 3:** Antioxidant capacity parameters obtained using several methods for studied samples.

Samples	IC_50_ (µg/mL)	TEAC (µmol Trolox/mg Plant Material)	HAPX (%)
*H. officinalis*	125.44 ± 4.70	57.39 ± 13.68	16.17 ± 3.58
*O. basilicum*	124.95 ± 4.46	25.69 ± 2.96	18.84 ± 1.12
*T. chamaedrys*	26.70 ± 0.96	87.77 ± 0.33	12.87 ± 3.35
Trolox	11.20 ± 0.20	–	–

Each value is the mean ± SD of three independent measurements.

The antioxidant activity of all three ethanol extracts was assessed by the DPPH radical bleaching method. Trolox (0.025 mg/mL) was used as the positive control (Table 3). The highest radical scavenging activity was showed by *T. chamaedrys* with IC_50_ = 26.70 ± 0.96 µg/mL, while the lowest was for the extracts of *O. basilicum* and *H. officinalis* which showed similar IC_50_ values (124.95 ± 4.46, and 125.44 ± 4.70 µg/mL respectively). The results suggest that there is not a statistically significant difference between hyssop and basil in terms radical scavenging activity (*p* = 0.902; *p* > 0.05), these species showing the lowest antioxidant effect. The IC_50(DPPH)_ values of the extracts increased in the following order: *T. chamaedrys* < *O. basilicum* < *H. officinalis. T. chamaedrys* showed a significantly higher antioxidant activity than *O. basilicum* and *H. officinalis* (*p* < 0.001). The lower the IC_50_ value means the more powerful the antioxidant capacity. According to this method, *T. chamaedrys* extract exhibited a high antioxidant capacity. The antioxidant activity value obtained showed that the extract of *T. chamaedrys* has a similar antioxidant potential to Trolox (*p* < 0.001). Comparing the antioxidant activities of T. *chamaerdys* L. var. *glanduliferum* from Serbia and *T. chamaedrys* from Romania, the ethanolic extract of the Romanian species (26.70 µg/mL) showed higher value than the aqueous and methanol extracts of the Serbian species (31.79, 29.46 µg/mL) [6,7]. However, the methanolic extract of *T. chamaedrys* from Turkey exhibited a strong antioxidant activity (18.00 μg/mg) [10]. The results for the ethanolic extract of *T. chamaedrys* were in good agreement with the phenolic compounds values listed in Table 2. Therefore, it is likely that the phenolic constituents present in these species are responsible for the antioxidant and free radical scavenging activities. Similar results were obtained earlier for the species of *H. officinalis* subsp. *angustifolius* from Turkey and for another sample of Romanian basil [13,14,28]. However, the H_2_O extract from *O. basilicum* from Serbia (17.93 μg/mL) showed more powerful antioxidant activity than Romanian basil [26].

The TEAC results are in reasonable agreement with the DPPH values while, surprisingly, the HAPX results are negatively correlated with both DPPH and TEAC (Table 3). The DPPH and TEAC assays are both based on the same principle (free radical scavenging) and all three assays (DDPH, TEAC and HAPX) present the same chemical mechanism (electron transfer) the notable difference is that in the case of the TEAC and HAPX, the solution is aqueous rather than ethanolic. The newly developed physiological relevant enzymatic assay (HAPX method) measures the capability of the extract components to quench the damage inflicted by hydrogen peroxide upon haemoglobin. This brings additional valuable information since it involves the interaction of the antioxidants molecules with a protein, *i.e.*, the physiological-relevant ferryl hemoglobin species (resulted by the action of hydrogen peroxide upon ferric hemoglobin). Even though the redox potential of the species is important in this interaction, the affinity and turn-over number of the haemoglobin towards different types of antioxidant molecules may be expected to be completely orthogonal factors, thus explaining the negative correlation. This reinforces the fact that the antioxidant activity of a given extract has different facets, depending on the condition and assay which is employed. The antioxidant activity values from Table 3 obtained by TEAC method showed that there is a highly significant difference between these three extracts (*p* < 0.001). The TEAC values increased in the following order: *T. chamaedrys* > *H. officinalis* > *O. basilicum*. The results obtained by HAPX assay (Table 3) suggest that there is not a statistically significant difference between the three extracts in terms of antioxidant activity (*p* > 0.05).

**Figure 4:**
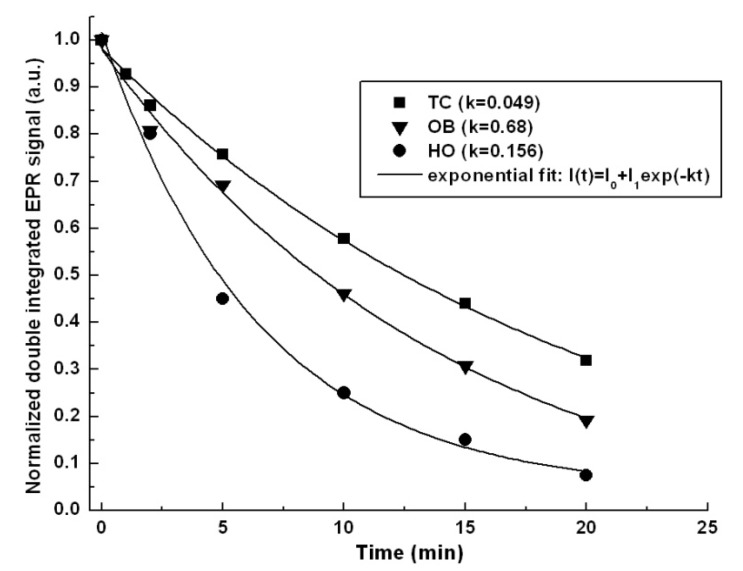
The rate of reaction between antioxidant compounds and TEMPO radical.

A simple method to study the qualitative antioxidant properties is EPR spectroscopy by using stable free radicals. In this paper, a commonly encountered nitrone spin probe, the nitroxidic Tempo ((2,2,6,6-tetramethylpiperidin-1-yl)oxyl) radical was used. The rate of reaction between antioxidant compounds and TEMPO radical was monitored by using normalized double integrated residual EPR signal which is correlated with the number of paramagnetic species in time (Figure 4).

The best fit was obtained using the first order exponential decay: I(t) = I_0_ + I_1_exp(‒kt), where I_0_ and I_1_ are the fit constants representing the double integral EPR signal intensity immediately after adding free radicals and after time t, respectively and k is the kinetic constant of the reaction corresponding to each type of extracts. The k constant is specific to each type of sample and processing way. It represents the redox rate of the Tempo radical in time and it is a fingerprint of the antioxidant compound quality. Comparing the calculated kinetic rates of the studied samples, one can observe that *H. officinalis* (HO) has the most significant antioxidant activity (k_HO_ = 0.156). It is also relevant to underline that it can be compared with the reference gallic acid, known to have an intense antioxidant activity, k = 0.16. *O. basilicum* (OB) and *T. chamaedrys* (TC) has also a relative moderate antioxidant capacity (k_OB_ = 0.068 and k_TC_ = 0.049). Take in account the kinetic constant values of each extract, the antioxidant capacity increased in the following order: *T. chamaedrys* < *O. basilicum* < *H. officinalis*.

### 2.4. Antimicrobial Activity

The ethanolic extracts of *H. officinalis*, *O. basilicum* and *T. chamaedrys* were investigated for their *in vitro* antimicrobial properties using a disk-diffusion method against a panel of microorganisms including *S. aureus*, *L. monocytogenes*, *E. coli*, *S. typhimurium* and *C. albicans.* After incubation, all plates were examined for any zones of growth inhibition, and the diameter of these zones were measured in millimeters (Table 4) [31,34,35].

**Table 4:** Results of the antimicrobial activity of *H. officinalis*, *O. basilicum* and * T. chamaedrys* extracts in agar diffusion method.

Samples	Zone of Inhibition (mm)				
	*Staphylococcus Aureus*	*Listeria Monocytogenes*	*Escherichia Coli*	*Salmonella Typhimurium*	*Candida Albicans*
*H. officinalis*	16.0 ± 0.07	12.0 ± 0.00	10.0 ± 0.05	10.0 ± 0.02	16 ± 0.05
*O. basilicum*	16.0 ± 0.05	11.0 ± 0.1	10.0 ± 0.05	11.0 ± 0.00	18 ± 0.1
*T. chamaedrys*	20 ± 0.1	15 ± 0.05	12 ± 0.15	11 ± 0.05	22 ± 0.00
Gentamicin	19 ± 0.05	18 ± 0.02	22 ± 0.00	18 ± 0.01	–
Fluconazole	–	–	–	–	25 ± 0.00

Notes: The values represent the average of three determinations ± standard deviations. Gentamicin (10 µg/disk) and Fluconazole (25 µg/well) were used as a positive control.

As it can be seen from the Table 4, all investigated plant extracts were active against all the microorganisms tested. The extracts of *H. officinalis* (4.66 ± 0.04 µg TPC/60 µL/disk) and *O. basilicum* (10.53 ± 0.17 µg TPC/60 µL/disk) showed a moderate antibacterial activity against *S. aureus* (inhibition diameter–16 mm), and low antibacterial effect on *L. monocytogenes*, *E. coli*, *S. typhimurium* (inhibition diameter between 10 and 15 mm). Similar results were obtained earlier for the species of *H. officinalis* subsp. *angustifolius* and *O. basilicum* from Turkey and India [13,22]. *T. chamaedrys* (14.62 ± 0.16 µg TPC/60 µL/disk) extract showed a stronger antibacterial activity against *S. aureus* (inhibition diameter–20 mm), than gentamicin used as reference antibiotic, and limited activity against the other bacteria tested. These herbal extracts were also active on *C. albicans* (the diameter of the zones of inhibition between 16 and 22 mm). Additionally, the extract of *T. chamaedrys* showed intensive activity against this fungal strain (inhibition diameter–22 mm), comparable to fluconazole. These results are in accordance with previous biological data for *T. species* from Serbia or Turkey (*T. chamaedrys*, *T. montanum*, *T. arduini*, *T. polium*) [5,10]. A one-way ANOVA test applied on the values from Table 4 (antimicrobial activity results) showed that the difference between these three extracts is statistically different for the all microbial strains (*p* < 0.001). The results of the present investigation suggest that *O. basilicum*, *H. officinalis* and *T. chamaedrys* exhibited an important antibacterial and antifungal activity. On the other hand the ethanolic extract of *T. chamaedrys* showed a remarkable antimicrobial activity, and among the microorganisms, the most sensitive was *S. aureus*.

## 3. Experimental

### 3.1. Plant Materials and Extraction Procedure

The aerial parts, in the blossom period, of the medicinal plants: *Ocimum basilicum* L. (Voucher No. 792) and *Hyssopus officinalis* L. (Voucher No. 781) from experimental fields of the University of Agricultural Sciences and Veterinary Medicine (Cluj-Napoca, Romania) and *Teucrium chamaedrys* L. (Voucher No. 1272), from the spontaneous flora (Valea Ariesului, Romania), were harvested in July 2013. Voucher specimens were deposited in the Herbarium of the Department of Pharmaceutical Botany of the “Iuliu Hatieganu” University of Medicine and Pharmacy, Cluj-Napoca, Romania. The plant materials were reduced to a proper degree of fineness. Twenty grams of each sample were weighed and extracted with 200 mL of 70% ethanol, for 30 min on a water bath, at 60 °C. The samples were then cooled down and centrifuged at 4500 rpm for 20 min, and the supernatants were recovered [31,36,37]. Stock standard solutions were prepared by accurately weighing 10 mg of chlorogenic, *p*-coumaric, caffeic, cichoric, caftaric, ferulic, sinapic, gentisic gallic acids, rutin, quercetin, isoquercitrin, quercitrin, hyperoside, kaempferol, myricetol, fisetin, patuletin, apigenin, luteolin, reference standards into separate 10 mL volumetric flasks and dissolving in methanol [28,29,30,31].

### 3.2. Chemicals and Instrumentation

Chlorogenic acid, *p*-coumaric acid, caffeic acid, rutin, apigenin, quercetin, isoquercitrin, quercitrin, hyperoside, kaempferol, myricetol, fisetin from Sigma (St. Louis, MO, USA), ferulic acid, sinapic acid, gentisic acid, gallic acid, patuletin, luteolin from Roth (Karlsruhe, Germany), cichoric acid, caftaric acid from Dalton (Toronto, ON, Canada). HPLC grade methanol, analytical grade orthophosphoric acid, hydrochloric acid and Folin-Ciocalteu reagent were purchased from Merck (Darmstadt, Germany), hydrogen peroxide, ABTS (2,2'-azinobis-3-ethylbenzotiazoline-6-sulphonic acid), sodium molybdate dihydrate, sodium nitrite, sodium hydroxide, sodium carbonate, sodium acetate trihydrate, and anhydrous aluminum chloride were from Sigma-Aldrich (Steinheim, Germany). Ethanol (Merck). DPPH (2,2-diphenyl-1-picrylhydrazyl) and Trolox (6-hydroxy-2,5,7,8-tetramethylchroman-2-carboxylic acid) were obtained from Alfa-Aesar (Karlsruhe, Germany). Bovine hemoglobin was purified following the general protocol of Antonini and Brunori [38]. The met forms of hemoglobin were prepared by ferricyanide treatment as previously described [39]. For the antimicrobial potential assaying of the plant extracts, all microorganism strains were distributed by MicroBioLogics^®^: *Staphylococcus aureus* ATCC 49444, *Listeria monocytogenes* ATCC 13076, *Escherichia coli* ATCC 25922, *Salmonella typhimurium* ATCC 14028 and one fungal strain, *Candida albicans* ATCC10231. All spectrophotometric data were acquired using a Jasco V-530 UV-vis spectrophotometer (Jasco International Co., Ltd., Tokyo, Japan).

### 3.3. HPLC-MS Analysis

#### Apparatus and Chromatographic Conditions

The detection and quantification of polyphenols was made in UV assisted by mass spectrometry detection: an Agilent Technologies 1100 HPLC Series system (Agilent, Santa Clara, CA, USA) equipped with G1322A degasser, G13311A binary gradient pump, column thermostat, G1313A autosampler and G1316A UV detector. The HPLC system was coupled with an Agilent 1100 Ion Trap SL mass spectrometer (LC/MSD Ion Trap VL) equipped with an electrospray or APCI ion source. For the separation, a reverse-phase analytical column was employed (Zorbax SB-C18 100 × 3.0 mm i.d., 3.5 μM particle); the work temperature was 48 °C. The detection of the compounds was performed on both UV and MS mode. The UV detector was set at 330 nm until 17.5 min, then at 370 nm. The MS system operated using an electrospray ion source in negative mode. The chromatographic data were processed using ChemStation and DataAnalysis software from Agilent [28,29,31].

The mobile phase was a binary gradient: methanol and acetic acid 0.1% (*v/v*). The elution started with a linear gradient, beginning with 5% methanol and ending at 42% methanol, for 35 min; then 42% methanol for the next 3 min [28,29,30,31].The flow rate was 1 mL/min and the injection volume was 5 µL. The MS signal was used only for qualitative analysis based on specific mass spectra of each polyphenol. The MS spectra obtained from a standard solution of polyphenols were integrated in a mass spectra library. Later, the MS traces/spectra of the analysed samples were compared to spectra from library, which allows positive identification of compounds, based on spectral mach. The UV trace was used for quantification of identified compounds from MS detection. Using the chromatographic conditions described above, the polyphenols eluted in less than 40 min (Table 5). Four polyphenols cannot be quantified in current chromatographic conditions due overlapping (caftaric acid with gentisic acid and caffeic acid with chlorogenic acid). However, all four compounds can be selectively identified in MS detection (qualitative analysis) based on differences between their molecular mass and MS spectra. For all compounds, the limit of quantification was 0.5 μg/mL, and the limit of detection was 0.1 μg/mL. The detection limits were calculated as minimal concentration producing a reproductive peak with a signal-to-noise ratio greater than three. Quantitative determinations were performed using an external standard method. Calibration curves in the 0.5–50 μg/mL range with good linearity (R^2^ > 0.999) for a five point plot were used to determine the concentration of polyphenols in plant samples [28,29,30,31]. The detection and quantification of polyphenols was performed in UV assisted by mass spectrometry detection. Due to peak overlapping, four polyphenol-carboxylic acids (caftaric, gentisic, caffeic, chlorogenic) were determined only based on MS spectra, whereas for the rest of the compounds the linearity of the calibration curves was very good (R^2^ > 0.998), with detection limits in the range of 18 to 92 ng/mL. The detection limits were calculated as the minimal concentration yielding a reproducible peak with a signal-to-noise ratio greater than three. Quantitative determinations were performed using an external standard method; retention times were determined with a standard deviation ranging from 0.04 to 0.19 min (Table 5). For all compounds, the accuracy was between 94.1.3% and 105.3%. Accuracy was checked by spiking samples with a solution containing each polyphenol in a 10 μg/mL concentration. In all analysed samples the compounds were identified by comparison of their retention times and recorded electrospray mass spectra with those of standards in the same chromatographic conditions. To avoid or limit the interference from background, the multiple reactions monitoring analysis mode was used instead of single ion monitoring (e.g., MS/MS instead of MS). The Agilent ChemStation (vA09.03) and DataAnalysis (v5.3) software were used for the acquisition and analysis of chromatographic data [28,29,31].

**Table 5:** Retention times (R_T_) of polyphenolic compounds (min).

Peak No.	Phenolic Compounds	*m/z*	R_T_ ± SD (min)	Peak No.	Phenolic Compounds	*m/z*	R_T_ ± SD (min)
1	Caftaric acid	311	3.54 ± 0.05	11	Rutin	609	20.20 ± 0.15
2	Gentisic acid	179	3.52 ± 0.04	12	Myricetin	317	21.13 ± 0.12
3	Caffeic acid	179	5.60 ± 0.04	13	Fisetin	285	22.91 ± 0.15
4	Chlorogenic acid	353	5.62 ± 0.05	14	Quercitrin	447	23.64 ± 0.13
5	*p*-Coumaric acid	163	9.48 ± 0.08	15	Quercetin	301	26.80 ± 0.15
6	Ferulic acid	193	12.8 ± 0.10	16	Patuletin	331	29.41 ± 0.12
7	Sinapic acid	223	15.00 ± 0.10	17	Luteolin	285	29.10 ± 0.19
8	Cichoric acid	473	15.96 ± 0.13	18	Kaempferol	285	32.48 ± 0.17
9	Hyperoside	463	18.60 ± 0.12	19	Apigenin	279	33.10 ± 0.15
10	Isoquercitrin	463	19.60 ± 0.10		Rosmarinic acid	360	20.8 ± 0.10

Note: SD, standard deviation.

### 3.4. Determination of Total Polyphenols, Caffeic Acid Derivatives and Flavonoids Content

TPC (total phenolic content) of the extracts were measured using the Folin-Ciocalteau method with some modifications [28,31,36,40,41,42]. Fifty µL of each ethanolic extract were mixed with Folin-Ciocalteu reagent (1.0 mL) and distilled water (10.0 mL) and diluted to 25.0 mL with a 290 g/L solution of sodium carbonate. The samples were incubated in the dark for 30 min. The absorbance was measured at 760 nm, using a JASCO UV-VIS spectrophotometer. Standard curve was prepared by using different concentrations of gallic acid and the absorbances were measured at 760 nm. TPC values were determined using an equation obtained from the calibration curve of gallic acid graph (R^2^ = 0.999). Total polyphenolic content was expressed as mg gallic acid/g dry material plant (mg GAE/g plant material).

The total flavonoid contents were determined and expressed as rutin as previously described in the Romanian Pharmacopoeia (X^th^ Edition, 1993) for *Cynarae folium* [37]. Each extract (5 mL) was mixed with sodium acetate (5.0 mL, 100 g/L), aluminum chloride (3.0 mL, 25 g/L), and made up to 25 mL in a calibrated flask with methanol. Each solution was compared with the same mixture without reagent. The absorbance was measured at 430 nm [16,37]. The total flavonoids content values was determined using an equation obtained from calibration curve of the rutin graph (R^2^ = 0.999).

The caffeic acid derivatives content in the plant materials was determined using the spectrophotometric method with Arnow’s reagent (10 g sodium nitrite and 10 g sodium molybdate made up to 100 mL with distilled water) [37]. The percentage of phenolic acids, expressed as caffeic acid equivalent on dry material plant (mg CAE/g plant material), was determined using an equation that was obtained from calibration curve of caffeic acid (R^2^ = 0.994). Each sample was analyzed in triplicate.

### 3.5. In Vitro Antioxidant Activity Assays

#### 3.5.1. DPPH Bleaching Assay

The DPPH assay provides an easy and rapid way to evaluate potential antioxidants. DPPH free radical method is an antioxidant assay based on electron-transfer that produces a violet solution in ethanol. This free radical, stable at room temperature is reduced in the presence of an antioxidant molecule, giving rise to a yellow solution. The free radical scavenging activity of the ethanolic extracts was measured in terms of hydrogen donating or radical scavenging ability using this method. Trolox was chosen as a standard antioxidant. The DPPH solution (0.1 g/L) in ethanol was prepared and 2.0 mL of this solution was added to 2.0 mL of extract solution (or standard) in ethanol at different concentrations (6.25–100 μg/mL). After 30 min of incubation at 40 °C in a thermostatic bath, the decrease in the absorbance (n = 3) was measured at 517 nm. The percent of DPPH discolouration was calculated as: DPPH scavenging ability = (A_control_ − A _sample_/A_control_) × 100, where Abs_control_ is the absorbance of DPPH radical + ethanol (containing all reagents except the sample) and Abs_sample_ is the absorbance of DPPH radical + sample extract. The control solution was prepared by mixing ethanol (2.0 mL) and DPPH radical solution (2.0 mL). Afterwards, a curve of % DPPH scavenging capacity *versus* concentration was plotted and IC_50_ values were calculated. IC_50_ denotes the concentration of sample required to scavenge 50% of DPPH free radicals [28,31,34,40,41,42]. The lower the IC_50_ value the more powerful the antioxidant capacity. If IC_50_ ≤ 50 µg/mL the sample has high antioxidant capacity, if 50 µg/mL < IC_50_ ≤ 200 µg/mL the sample has moderate antioxidant capacity and if IC_50_ > 200 µg/mL the sample has no relevant antioxidant capacity [43].

#### 3.5.2. TEAC Assay (Trolox Equivalent Antioxidant Capacity)

The TEAC assay or 2,2'-azinobis-3-ethylbenzotiazoline-6-sulphonic acid (ABTS) assay is based on scavenging of the ABTS^+^ radical cation by the antioxidants present in a sample. In a quartz cuvette, to 955 μL of PBS (phosphate buffer saline) the following were added: 20 μL of ethanolic extracts of *H. officinalis, O. basilicum* and *T. chamaedrys* (diluted 100 times), and 25 μL of ABTS^+^ (from 74 mM stock solution). The experiments were done in triplicate, with a relative standard deviation of less than 6%. The plant extract in the assay mixture was 8.24 mg/L. The content of the generated ABTS^+^ radical was measured at 734 nm after 600 s reaction time and was converted in Trolox equivalents by the use of a calibration curve (R^2^ = 0.998) constructed with 0, 2, 4, 6, 8, 10 mg/L. Trolox standards [31,44].

#### 3.5.3. Hemoglobin/Ascorbate Peroxidase Activity Inhibition (HAPX) Assay

Bovine hemoglobin was purified following the general protocol of Antonini and Brunori [38]. Hemoglobin ascorbate peroxidase activity has previously been described in detail [45]. In a quartz cuvette 50 mM sodium acetate buffer (956 μL, pH 5.5), were mixed with ascorbic acid (7 μL, 50 mM), hydrogen peroxide (20 μL, 50 mM) and 10-times diluted extracts of *H. officinalis*, *O. basilicum* and *T. chamaedrys* (10 μL, final concentration of 82.4 mg/L). After 12–15 s, met-hemoglobin (met-Hb, 7 μL) from a stock solution of 1.4 mM was added to the reaction mixture and the 290 nm absorbance was further monitored. A measurable significant inhibition of the ascorbic acid consumption was observed compared to the reference (run in four different experiments) in which the extract was replaced by an equal amount of extraction solvent [31,45]. The slope of each sample was calculated at the tested concentration and also without the tried sample (blank). The inhibition of the ascorbic acid consumption was determined as follows: HAPX = 100 − [(slope of the sample/slope of the blank) × 100].

#### 3.5.4. EPR Measurements

The EPR spectra were measured using an EMX Micro spectrometer (Bruker BioSpin GmbH, Rheinstetten, Germany). EPR instrument conditions were as follows: microwave frequency 9.43 GHz, microwave power 15.89 mW, modulation frequency 100 kHz, modulation amplitude 3 G, sweep rate 10 G/s; time constant 10.24 ms, average of three sweeps for each spectrum, room temperature. For the TEMPO ((2,2,6,6-tetramethylpiperidin-1-yl)oxyl radical) scavenging by the extract and EPR monitored, 20 µL of 3.43 mM TEMPO is quickly mixed with 30 µL of extract and transferred with a syringe into an EPR micro tube. The EPR signal is registered at defined time interval and the double integrals are calculated. The kinetic profile obtained is fitted with a first order exponential decay function and the kinetic constant is considered an antioxidant parameter [46].

### 3.6. Determination of Antimicrobial Activity

The disc-diffusion assay was used to determine the antimicrobial activity of the investigated ethanolic extracts of *O. basilicum*, *H. officinalis* and *T. chamaedrys* against a panel of microorganisms including two gram-positive bacteria *Staphylococcus aureus* (ATCC 49444), and *Listeria monocytogenes* (ATCC 13076)*,* two gram-negative bacteria, *Salmonella typhimurium* (ATCC 14028) and *Escherichia coli* (ATCC 25922)*,* and the fungus Candida albicans (ATCC10231) [35]. Each microorganism was suspended in Mueller Hinton (MH) broth and diluted approximately to 10E6 colony forming unit (cfu)/mL. They were “flood-inoculated” onto the surface of MH agar and MH Dextroxe Agar (MDA) and then dried. Six-millimeter diameter wells were cut from the agar using a sterile cork-borer, and 60 μL of each extract were delivered into the wells. The plates were incubated at 37 °C and the diameters of the growth inhibition zones were measured after 24 h. Gentamicin (10 µg/well) and fluconazole (25 µg/well) were used as standard drugs. The controls were performed with only sterile broth and with only overnight culture and 10 μL of 70% ethanol. All tests were performed in triplicate, and clear halos greater than 10 mm were considered as positive results.

### 3.7. Statistical Analysis

A statistical approach was designed and the experimental data were evaluated using one-way analysis of variance (ANOVA), with *p* < 0.05 as threshold for statistical significance. The statistical results confirm the hypothesis that the differences between the results are either not significant (*p* > 0.05), significant (0.001 < *p* < 0.05) or highly significant (*p* < 0.001). The average of multiple measurements (triplicates or more) was listed in the tables together with the standard deviations. Statistical analysis was performed using Excel software package.

## 4. Conclusions

We have determined the polyphenolic composition, the antioxidant and antimicrobial activities for *O. basilicum*, *H. officinalis* and *T. chamaedrys* from Romania, for their better pharmacognostical and phytochemical characterisation. The comparative phytochemical study showed qualitative and quantitative differences between the three *Lamiaceae* species; *T. chamaedrys* was the richest one concerning polyphenolic compounds. The antioxidant activity evaluated using the DPPH bleaching method, TEAC assay, and an EPR spectroscopy method indicated that *T. chamaedrys* extract was the most powerful antioxidant, related with the polyphenolic total content. The antimicrobial tests underlined an important activity against *Staphylococcus aureus* and *Candida albicans* for all samples. Our results confirm that *H. officinalis, O. basilicum* and *T. chamaedrys* may be considered a potential source of polyphenols with antioxidant and antimicrobial properties. Further studies of these medicinal plants should be directed to carry out *in vivo* studies in order to prepare natural pharmaceutical products of high value.
